# Supplementary material for: Diabetes Capabilities for the Healthcare Workforce Identified via a 3-Staged Modified Delphi Technique
Source: Int J Environ Res Public Health. 2022 Jan 17;19(2):1012. doi: 10.3390/ijerph19021012 (PMC8775604; doi:10.3390/ijerph19021012)
Supplement: Supplementary file 1 [file ijerph-19-01012-s001.zip › ijerph-1536577-supplementary.pdf]

Table S1: Expert Advisory Group members' characteristics

Qualifications and roles	Current employer
PhD, Advanced Practising Dietitian, Consumer and Academic.	University–Human nutrition
Master in Nursing, Nurse Practitioner, Midwife, CDE.<br>Held roles in academia as course coordinator for diabetes education degrees and leadership roles in Australian Diabetes Educators Association (ADEA) and the Australian Diabetes in Pregnancy Society.	District health service<br>Private practice
Senior Clinical Nurse Adviser, CDE<br>Held leadership roles nationally in ADEA.	Not-for-profit aged care service and healthcare provider / Private practice
PhD, BA(Psych), Post-doctoral Research Fellow and Behavioural Scientist.	University–Behavioural science
CDE, BA, Lecturer, unit chair for a graduate certificate of diabetes education and Academic.	University–Nursing

Table S2: Characteristics of participants in study

	Pilot and EAG (*n*=15)		Consultation Group (*n*=15)		Delphi 1 Round 1 (*n*=50)		Delphi 1 Round 2 (*n*=44)		Delphi 2 Round 1 (*n*=40)		Delphi 2 Round 2 (*n*=37)		Focus Group (*n*=4)	
**Discipline**	*n*	%	*n*	%	*n*	%	*n*	%	*n*	%	*n*	%	*n*	%
Aboriginal & Torres Strait Islander Health Worker	0	0	0	0	0	0	0	0	0	0	0	0	0	0
Dietitian	2	13	3	20	5	10	5	11	5	13	5	14	1	25
Exercise physiologist	0	0	0	0	1	2	1	2	1	3	1	3	0	0
Nurse	10	67	8	53	39	78	33	75	29	73	27	73	2	50
Physiotherapist	0	0	0	0	0	0	0	0	0	0	0	0	0	0
Pharmacist	0	0	4	27	2	4	2	5	2	5	2	5	1	25
Podiatrist	1	7	0	0	2	4	2	5	2	5	2	5	0	0
Psychologist/Behavioural	2	13	0	0	1	2	1	2	1	3	0	0	0	0
**Gender**	*n*	%	*n*	%	*n*	%	*n*	%	*n*	%	*n*	%	*n*	%
Female	15	100	14	93	49	98	43	98	39	97	37	100	4	100
Male	0	0	1	7	1	2	1	2	1	3	0	0	0	0
**Years of practice**	*n*	%	*n*	%	*n*	%	*n*	%	*n*	%	*n*	%	*n*	%
> 5 - 10 years	0	0	0	0	4	8	4	9	4	10	4	11	0	0
>10 - 15 years	2	13	3	20	2	4	2	5	2	5	2	5	0	0
> 15 - 20 years	0	0	0	0	8	16	8	18	8	20	6	16	0	0
> 20 years	6	40	2	13	6	12	4	9	3	8	4	11	2	50
> 30 years	7	47	10	67	30	60	26	59	23	58	21	57	2	50
**State/territory of practice**	*n*	%	*n*	%	*n*	%	*n*	%	*n*	%	*n*	%	*n*	%
ACT / NSW	3	20	3	20	14	28	11	25	10	25	7	19	1	25
Northern Territory	0	0	1	7	2	4	2	5	2	5	2	5	0	0
Queensland	0	0	1	7	6	12	6	14	6	15	6	16	1	25
South Australia	0	0	3	20	4	8	4	9	3	8	2	5	1	25
Tasmania	4	27	1	7	3	6	3	7	3	8	2	5	0	0
Victoria	8	53	4	27	15	30	13	30	11	28	12	32	1	25
Western Australia	0	0	2	13	6	12	5	11	5	13	6	16	0	0
**Employment locality (all)**	*n*	%	*n*	%	*n*	%	*n*	%	*n*	%	*n*	%	*n*	%
RA1 – Major cities	3	20		33		32				31			2	50
RA2 – Inner regional	4	27		20		27				28				
RA3 – Outer regional	6	40		27		19				19			2	50
RA4 – Remote	2	13		13		12				11				
RA5 – Very remote	0	0		7		10				11				
**Age group**	*n*	%	*n*	%	*n*	%	*n*	%	*n*	%	*n*	%	*n*	%
< 30 years	2	13	0	0	1	2	1	2	1	3	1	3	0	0
> 30 - 50 years	9	60	4	27	14	28	11	25	10	25	9	24	1	25
> 50 years	4	27	11	73	35	70	32	73	29	73	27	73	3	75
**Employment sector**	*n*	%	*n*	%	*n*	%	*n*	%	*n*	%	*n*	%	*n*	%
Primary (including community)	2	13		25		25				28			1	25
Tertiary/Hospital (inc out-pts)	5	34		52		47				54			2	50
Research/University	6	40		15		18				13			0	0
Private practice	2	13		8		10				5			1	25
**Highest qualification**	*n*	%	*n*	%	*n*	%	*n*	%	*n*	%	*n*	%	*n*	%
General nurse training (Hosp)	0	0	0	0	2	4			1	3			0	0
Bachelor's degree	0	0	0	0	3	6			1	3			0	0
Postgraduate Degree	6	40	11	73	29	58			25	63			3	75
Clinical Masters	2	13	1	7	10	20			7	18			0	0
Masters (Research)	1	7	3	20	4	8			4	10			1	25
PhD or Doctorate	6	40	0	0	2	4			2	5			0	0
**Degree in diabetes**			*n*	%	*n*	%							*n*	%
Yes postgrad			15	100	42	84							3	75
**Involved with medicines**					*n*	%							*n*	%
Yes					39	78							3	75
Maybe					5	10							1	25
No					6	12							0	0

Figure S1: Delphi survey response rate

PHASE 1 - Round 1

57 participants invited

52 started the survey

- 41 completed the survey
- 6 partially completed the survey including Delphi questions
- 2 only consented and answered several demographic questions*
- 3 did not complete all the research questions
- 5 did not start the survey

50/57 (88%)

PHASE 1 - Round 2

48 participants invited

44 started the survey

- 41 completed the survey
- 3 partially completed the survey
- 3 opted out
- 1 overseas
- 2 did not start the survey

44/50 (88%)

44/48 (91.7%)

PHASE 2 - Round 1

48 participants invited

40 started the survey

- 36 completed the survey
- 4 partially completed the survey
- 2 opted out
- 6 did not start the survey

40/50 (80%)

40/48 (83.3%)

PHASE 2 - Round 2

46 participants invited

37 started the survey

- 36 completed the survey
- 1 partially completed the survey
- 2 opted out
- 7 did not start the survey

37/50 (74%)

37/46 (80.4%)

Table S3: Ranking of practice level models by Delphi participants

Framework	Rank No. 1		Rank No. 2		Rank No. 3		Rank No. 4					Total
	%	No.	%	No.	%	No.	%	No.	Mean	Medium	MAD	No.
Model 1	32.6%	14	23.3%	10	16.3%	7	27.9%	12	2.40	2	1.0	43
Model 2	30.2%	13	20.9%	9	34.9%	15	14.0%	6	2.33	2	0.9	43
Model 3	30.2%	13	48.8%	21	16.3%	7	4.7%	2	1.95	2	0.6	43
Model 4	7.0%	3	7.0%	3	32.6%	14	53.5%	23	3.33	4	0.7	43

NB: One participant answered all the questions but did not fully complete the ranking; therefore, this individual's responses were not included.

Figure S2: Model 3 Multidisciplinary

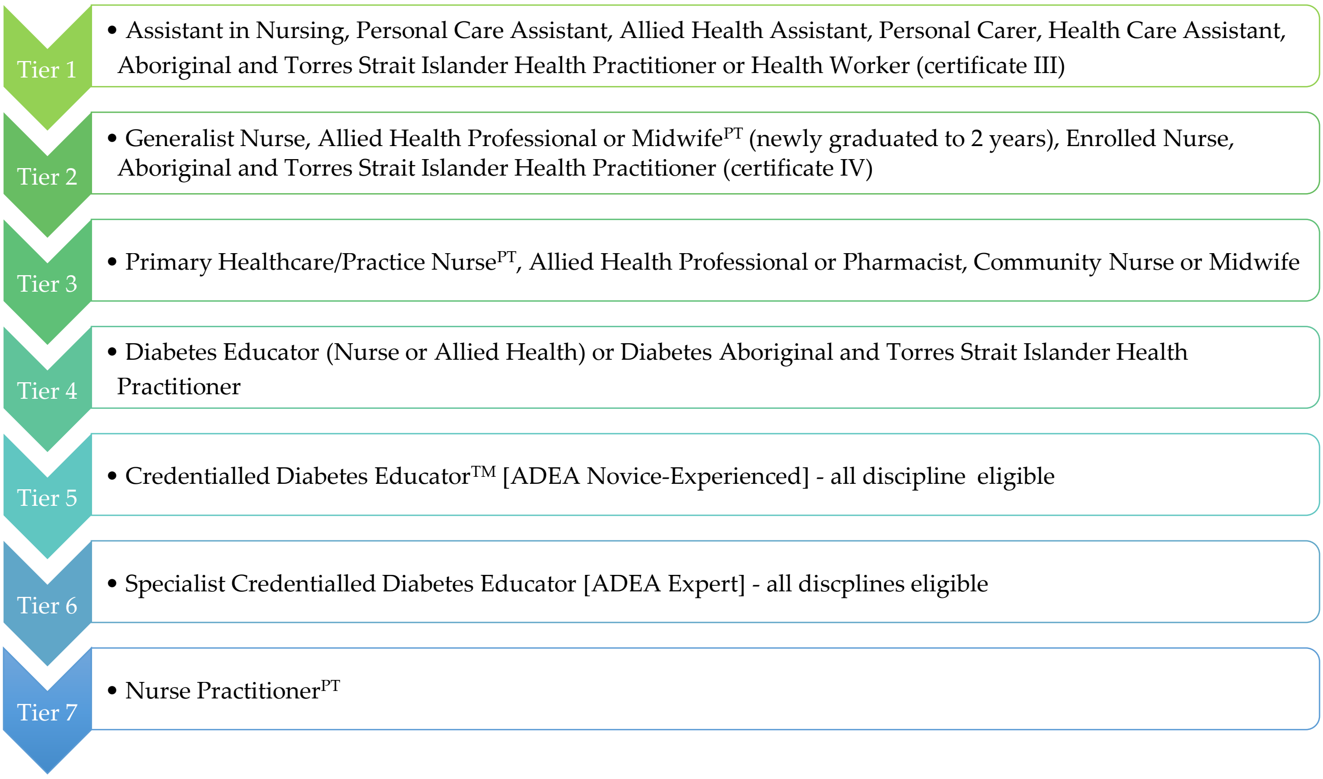

NB: Advanced Management Credentialled Diabetes Educator with non-medical prescriber endorsement recommended and was presented in practice level 7. Removed on advice that the role does not currently exist and could more likely sit in practice level 6 dependent on scope and training.

Table S4: Stages of diabetes clinical competence associated with practice levels

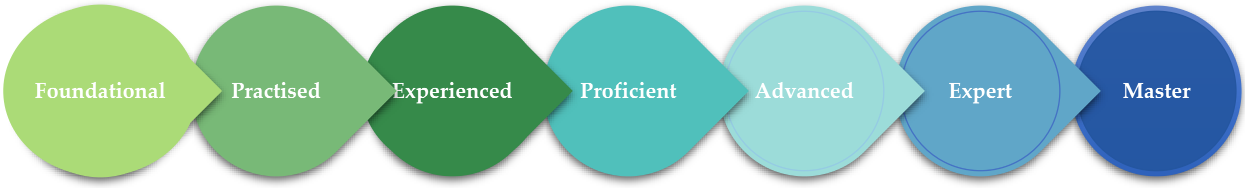

Foundational level (Practice level 1): the healthcare provider who works at a foundational level provides care to a wide variety of consumers with a broad range of medical conditions. They have some basic knowledge and skills to support diabetes care under direction and close supervision within routine tasks and procedures.

Practised level (Practice level 2): the healthcare provider who works at a practised level provides care to varied consumer groups and a broad range of conditions. The generalist healthcare provider with a broad scope of practice is involved in aspects of diabetes care and has basic diabetes care skills.

Experienced level (Practice level 3): the healthcare provider who works at an experienced level provides care to a broad consumer group who have complex healthcare needs and a broad range of conditions. The experienced generalist healthcare provider has gained more focused involvement to aspects of diabetes care and has started or needs to work towards developing competencies in diabetes over several years.

Proficient level (Practice level 4): the healthcare provider who works at a proficient level provides diabetes care to a varied consumer group with a limited range of medical conditions, primarily diabetes. Their scope of practice is narrowing, and they use advanced skills to promote shared decision-making with people living with diabetes.

Advanced level (Practice level 5): the healthcare provider who works at an advanced level has advanced competencies in diabetes care. They extend and develop their competence through mentoring and extensive experience in the speciality area of diabetes. Their scope of practice is focused on diabetes with a varied and broad diabetes consumer group.

Expert level (Practice level 6): the advanced practice healthcare provider who works at an expert level to provide care to a specific diabetes consumer group, e.g. paediatrics or pregnancy. The healthcare provider functions at a high advanced practice level and focuses on a speciality area in diabetes. Their scope of practice and diabetes consumer group are narrow, and may include non-medical prescribing skills.

Master level (Practice level 7): the Master healthcare provider uses intuitive reflectivity in complex care and has mastered many health and diabetes care elements. They have a broad set of highly advanced practice assessment and clinical skills to complement a narrow scope of practice. They identify and utilise expertise for specific complex consumer cases and work within a defined scope of practice and consumer group.

Table S5: Attributes required that underpin capabilities

Attributes to support excellent communication, collaboration and advocacy	Attributes to strive for excellence	Attributes to ensure the professional's health and wellbeing to enable their adaptability to dynamic environments
- active listener<br>- caring<br>- collaborative<br>- compassion<br>- cultural competence<br>- empathy<br>- inclusiveness<br>- non-judgemental<br>- openness<br>- personable<br>- willingness to learn from the consumer	- analytical<br>- adaptable<br>- accountability<br>- confidence<br>- commitment<br>- creativity<br>- ethical<br>- honesty<br>- initiative<br>- inspiring<br>- integrity<br>- life-long learner<br>- open-mindedness<br>- professionalism<br>- reflective	- mindfulness<br>- self-awareness<br>- self-concept<br>- self-knowledge<br>- self-management

Table S6: Ranking and central tendencies scores for capability components for practice levels

The health professional or health assistant will:

Display clinical assessment capacity	Round 1				Round 2			
**Capability components**	Rank %	Mean (Med)	MAD	Out come	Rank %	Mean (Med)	MAD	Out come
**PRACTICE LEVEL 1 - FOUNDATIONAL**								
Demonstrate foundational clinical measurement skills	58	3.63(4.0)	1.03	R2	81	3.97(4.0)	0.88	✓
Support awareness of risk factors for diabetes and its care	75	3.88(4.0)	0.74	✓				
**PRACTICE LEVEL 2 - PRACTISED**								
Demonstrate sound clinical assessment skills	95	4.58(5.0)	0.53	✓				
Assess for diabetes risk factors	87	4.32(4.0)	0.65	✓				
**PRACTICE LEVEL 3 – EXPERIENCED**								
Demonstrate sound clinical assessment skills	97	4.66(5.0)	0.47	✓				
Display a sound understanding of diabetes risk factors & impact on Aboriginal and Torres Strait Islander people	95	4.66(5.0)	0.49	✓				

Exemplify clinical assessment capacities	Round 1				Round 2			
**Capability components**	Rank %	Mean (Med)	MAD	Out come	Rank %	Mean (Med)	MAD	Out come
**PRACTICE LEVEL 4 – PROFICIENT**								
Demonstrate developed advanced clinical assessment skills in diabetes	92	4.61(5.0)	0.54	✓				
Campaign for early screening and diabetes prevention	95	4.66(5.0)	0.49	✓				
**PRACTICE LEVEL 5A – ADVANCED**								
Demonstrate developed advanced clinical assessment skills in diabetes	95	4.61(5.0)	0.52	✓				
Campaign for early screening and diabetes prevention	97	4.66(5.0)	0.47	✓				
**PRACTICE LEVEL 5B – ADVANCED**								
Demonstrate proven advanced clinical assessment skills in diabetes	97	4.82(5.0)	0.31	✓				
Campaign for earlier detection of type 1 diabetes and type 2 diabetes	92	4.63(5.0)	0.52	✓				
**PRACTICE LEVEL 6 – EXPERT**								
Illustrate comprehensive assessment skills in their specialty area	97	4.87(5.0)	0.24	✓				
**PRACTICE LEVEL 7 - MASTER**								
Perform comprehensive assessments in complex diabetes cases [Proposed advanced practice CDE]	97	4.89(5.0)	0.19	✓				
Adeptly perform comprehensive assessments in complex diabetes cases [Nurse practitioner]	97	4.89(5.0)	0.19	✓				
Demonstrate a high level of clinical proficiency in complex situations [Nurse practitioner]	97	4.89(5.0)	0.19	✓				

The health professional or health assistant will:

Support diabetes self-management education (DSME)	Round 1				Round 2			
**Capability components**	Rank %	Mean (Med)	MAD	Out come	Rank %	Mean (Med)	MAD	Out come
**PRACTICE LEVEL 1 - FOUNDATIONAL**								
Promote foundational diabetes self-management education	48	3.50(3.0)	0.83	R2	53	3.44(4.0)	0.81	**FG-X**
Encourage early diabetes prevention and screening	70	3.88(4.0)	0.88	R2	53	3.42(4.0)	1.00	**FG-✓**
~~Reinforce~~ *Demonstrate* awareness of foundational healthy eating and physical activity intervention	73	3.90(4.0)	0.75	R2	61	3.64(4.0)	0.72	**FG-✓***
Monitor for hypoglycaemia and hyperglycaemia	65	3.78(3.8)	0.84	R2	81	4.00(4.0)	0.61	✓
Describe and support screening for diabetes-related complications	30	2.95(3.0)	0.77	**X**				
Support care of the person with established cardiovascular disease	38	3.05(3.0)	0.76	**X**				

~~Promote~~ *Participate* in quality diabetes care in residential-aged-care and end-of-life stages	60	3.73(4.0)	0.88	R2	53	3.58(4.0)	0.88	**FG-**✓*
Demonstrate awareness of healthcare needs of people living with an intellectual disability								**FG**-Add
**PRACTICE LEVEL 2 - PRACTISED**								
Demonstrate a sound knowledge of the pathophysiology of diabetes	63	3.79(4.0)	0.74	R2	78	4.08(4.0)	0.56	✓
Apply chronic conditions self-management principles and education strategies	74	4.03(4.0)	0.67	✓				
Support diabetes prevention and early screening	76	4.11(4.0)	0.71	✓				
Reinforce healthy eating and physical activity intervention	87	4.24(4.0)	0.56	✓				
Monitor and manage glycaemic emergencies	82	4.34(5.0)	0.80	✓				
Assess for sick day management skills	55	3.66(4.0)	0.96	R2	44	3.53(3.0)	0.81	**FG*-**✓*
Advocate for and support appropriate diabetes care for people undergoing surgical procedures to enable quality in-hospital care	84	4.18(4.0)	0.64	✓				
Guide screening for diabetes-related complications	50	3.55(3.5)	0.71	R2	47	3.42(3.0)	0.72	**FG-**✓
Promote screening for gestational diabetes mellitus	90	4.39(4.5)	0.61	✓				
Support and advocate for pre-conception care and pregnancy planning in people living with prediabetes or diabetes	84	4.21(4.0)	0.62	✓				
Awareness of the impact of both diabetes in pregnancy and of pregnancy on diabetes	84	4.24(4.0)	0.64	✓				
Engage in antenatal and postnatal care related to a pregnancy complicated by diabetes	68	3.92(4.0)	0.74	R2	70	3.83(4.0)	0.73	**FG-**✓
Assess for healthy growth and development in children and adolescents living with diabetes	74	3.97(4.0)	0.57	✓				
Promote health and wellbeing in children and adolescents living with diabetes	79	4.16(4.0)	0.62	✓				
Demonstrate awareness of and monitors and educate the person with established cardiovascular disease	66	3.82(4.0)	0.77	R2	64	3.78(4.0)	0.62	**FG-**✓
~~Drive quality~~ *Promote* improved diabetes care in residential-aged-care and end-of-life stages	71	4.00(4.0)	0.74	R2	67	3.86(4.0)	0.69	**FG-**✓*
**PRACTICE LEVEL 3 – EXPERIENCED**								
Demonstrate a sound knowledge of the pathophysiology of diabetes	90	4.29(4.0)	0.56	✓				
Apply chronic conditions self-management principles and education strategies	97	4.53(5.0)	0.55	✓				
Apply evidence-based and self-management education, intervention and techniques to improve outcomes for people living with prediabetes and diabetes	90	4.32(4.0)	0.65	✓				
Drive early diabetes prevention and screening	87	4.39(5.0)	0.67	✓				
Explain healthy eating and physical activity intervention	87	4.34(5.0)	0.69	✓				
Educate about, monitor and manage glycaemic emergencies	82	4.16(4.0)	0.71	✓				
Promote sick day self-management skills	68	3.89(4.0)	0.78	R2	78	3.96(4.0)	0.53	✓
Guide screening for diabetes-related complications	84	4.24(4.0)	0.72	✓				
Monitor and educate the person with established cardiovascular disease	84	4.08(4.0)	0.58	✓				
Drive quality diabetes care in residential-aged-care and end-of-life stages	79	4.16(4.0)	0.75	✓				
Demonstrate cultural competence and advocate for people of Aboriginal and Torres Strait Islander descent	97	4.71(5.0)	0.43	✓				

**Shapes DSME, support and care**	Round 1				Round 2			
**Capability components**	Rank %	Mean (Med)	MAD	Out come	Rank %	Mean (Med)	MAD	Out come
**PRACTICE LEVEL 4 – PROFICIENT**								
Demonstrate a thorough understanding of the pathophysiology of main diabetes types	97	4.74(5.0)	0.40	✓				

Engage in the advanced application of chronic conditions self-management principles	97	4.71(5.0)	0.43	✓	
Identify vulnerable populations and considers the impact of coexisting healthcare conditions	97	4.66(5.0)	0.47	✓	
Educate in healthy eating and physical activity intervention	95	4.79(5.0)	0.35	✓	
Display advanced skills in monitoring and managing glycaemic emergencies	95	4.68(5.0)	0.47	✓	
Display basic sick day management skills	97	4.82(5.0)	0.31	✓	
Promote knowledge of and screening for diabetes-related complications	97	4.79(5.0)	0.34	✓	
Promote quality diabetes care in residential-aged-care and end-of-life stages	95	4.53(5.0)	0.55	✓	
Demonstrate an advanced understanding of the importance of managing diabetes in acute care or hospital setting	90	4.45(5.0)	0.67	✓	
**PRACTICE LEVEL 5A – ADVANCED**					
Demonstrate an advanced understanding of the pathophysiology of the major diabetes types	97	4.79(5.0)	0.34	✓	
Adapt to appropriate chronic condition self-management strategies for presentation	97	4.74(5.0)	0.40	✓	
Reduce the impact of diabetes on people from vulnerable groups with competing healthcare conditions	95	4.61(5.0)	0.52	✓	
Integrate an advanced understanding of healthy eating and physical activity interventions	97	4.74(5.0)	0.40	✓	
Provide education in and promote best practice care of people living with diabetes and prediabetes	97	4.79(5.0)	0.34	✓	
Apply health education skills to improve outcomes for people living with diabetes	97	4.74(5.0)	0.40	✓	
Demonstrate advanced skills in monitoring and managing glycaemic emergencies	97	4.66(5.0)	0.47	✓	
Demonstrate advanced sick day management skills	97	4.61(5.0)	0.50	✓	
Display advanced understanding of diabetes-related complications and their management	97	4.63(5.0)	0.48	✓	
Display advanced understanding of attributes of quality diabetes care in residential-aged-care and end-of-life stages	97	4.53(5.0)	0.52	✓	
Advise of important principles for quality care of people living with diabetes in the in-hospital setting	95	4.37(4.0)	0.57	✓	
**PRACTICE LEVEL 5B – ADVANCED**					
Demonstrate a comprehensive understanding of the pathophysiology of all types of diabetes	97	4.82(5.0)	0.31	✓	
Promote the use of chronic conditions self-management principles	97	4.84(5.0)	0.27	✓	
Manage complex diabetes impacted by coexisting conditions in vulnerable groups	97	4.68(5.0)	0.45	✓	
Provide an advanced explanation of healthy eating and physical activity interventions	97	4.76(5.0)	0.37	✓	
Provide structured education in and consults on best practice management and care of people living with diabetes and prediabetes	97	4.76(5.0)	0.37	✓	
Act as a health education consultant	95	4.55(5.0)	0.54	✓	
Consult in monitoring and managing glycaemic emergencies	97	4.79(5.0)	0.34	✓	
Consult in sick day management	97	4.84(5.0)	0.27	✓	
Act as a consultant for the management of diabetes-related complications	95	4.74(5.0)	0.42	✓	
Act as a consultant for quality diabetes care in residential-aged-care and end-of-life stages	95	4.63(5.0)	0.50	✓	
Act as a consultant for managing people living with diabetes in acute or hospital settings	92	4.61(5.0)	0.54	✓	

**PRACTICE LEVEL 6 – EXPERT**					
Provide education in and promote best practice management and care of people living with diabetes and prediabetes	97	4.84(5.0)	0.27	✓	
Demonstrate expertise in diabetes health education to improve outcomes for people living with diabetes	97	4.84(5.0)	0.27	✓	
Expertly monitor and manage glycaemic emergencies	95	4.82(5.0)	0.32	✓	
Develop personalised diabetes care related to healthy eating and, recreational activity and physical activity programs	95	4.82(5.0)	0.32	✓	
Expertly manage sick days in people living with diabetes	95	4.84(5.0)	0.28	✓	
Expertly screen or direct screening for diabetes-related complications	92	4.71(5.0)	0.46	✓	
Expertly support and manage gestational diabetes mellitus	97	4.71(5.0)	0.43	✓	
Lead pre-conception care and pregnancy planning in women with diabetes or prediabetes	95	4.61(5.0)	0.52	✓	
Proficiently provide and coordinate ante- and postnatal care related to pre-existing diabetes	95	4.63(5.0)	0.50	✓	
Advocates for quality diabetes care in pregnancy	97	4.82(5.0)	0.31	✓	
Proficiently provides care for healthy growth & development, and health and wellbeing in paediatrics and adolescents	97	4.66(5.0)	0.47	✓	
Astute to other issues in paediatrics and youth that impact diabetes care and coordinates care	94	4.66(5.0)	0.47	✓	
Leads transition of care from paediatric to adult clinics	95	4.71(5.0)	0.44	✓	
Coordinate and lead complex diabetes impacted by coexisting conditions or circumstances in vulnerable groups	95	4.68(5.0)	0.47	✓	
Provide expert advice on the care of people living with diabetes in the acute or in-hospital setting	95	4.74(5.0)	0.42	✓	
Demonstrate expertise in diabetes care in residential-aged-care and end-of-life stages	97	4.82(5.0)	0.31	✓	
**PRACTICE LEVEL 7 - MASTER**					
Display an advanced expert understanding of the implication of all diabetes type	97	4.82(5.0)	0.31	✓	
Recognise the complexity of clinical care and responds to high-risk diabetes needs	97	4.84(5.0)	0.27	✓	
Educate & support others to enable their active participation in care	95	4.76(5.0)	0.40	✓	
Demonstrate advanced clinical leadership and management skills	95	4.76(5.0)	0.40	✓	
Advocates for, participates in, or leads systems that support safe care, partnerships and professional growth	95	4.74(5.0)	0.43	✓	
Integrate and advocates for specialised knowledge to support diabetes care in all vulnerable groups	97	4.79(5.0)	0.34	✓	
Expertly support and manage gestational diabetes mellitus	97	4.87(5.0)	0.24	✓	
Lead pre-conception care and pregnancy planning in women with diabetes or prediabetes	95	4.76(5.0)	0.39	✓	
Proficiently provide and coordinate ante- and postnatal care related to pre-existing diabetes	97	4.79(5.0)	0.34	✓	
Advocates for quality diabetes care in pregnancy	97	4.84(5.0)	0.27	✓	
Proficiently provides care for healthy growth and development, and health and wellbeing in paediatrics and adolescents	97	4.79(5.0)	0.34	✓	
Astute to other issues in paediatrics and youth that impact diabetes care and coordinates care	97	4.74(5.0)	0.40	✓	
Leads transition of care from paediatric to adult clinics	97	4.82(5.0)	0.31	✓	
Coordinate and lead complex diabetes impacted by coexisting conditions or circumstances in vulnerable groups	97	4.82(5.0)	0.31	✓	
Provide expert advice on the care of people living with diabetes in the acute or in-hospital setting	95	4.79(5.0)	0.35	✓	
Demonstrate expertise in diabetes care in residential-aged-care and end-of-life stages	97	4.82(5.0)	0.31	✓	

The health professional or health assistant will:

Build therapeutic relationships	Round 1				Round 2			
Capability components	Rank %	Mean (Med)	MAD	Out come	Rank %	Mean (Med)	MAD	Out come
**PRACTICE LEVEL 1 - FOUNDATIONAL**								
Support the person living with diabetes to identify solutions to improve health	50	3.43(3.5)	0.78	R2	50	3.42(3.5)	0.75	**FG-✓**
Value and respect diversity	98	4.60(5.0)	0.50	✓				
Be culturally sensitive	98	4.68(5.0)	0.47	✓				
**PRACTICE LEVEL 2 - PRACTISED**								
Acknowledge, understand and respond to the needs of others	97	4.63(5.0)	0.48	✓				
Respect the informed health care decision of the person living with diabetes	100	4.71(5.0)	0.41	✓				
Incorporate cultural and diversity considerations into own practice	100	4.71(5.0)	0.41	✓				
Understand the importance of self-awareness in caring work	100	4.63(5.0)	0.47	✓				
**PRACTICE LEVEL 3 – EXPERIENCED**								
Recognise the emotions and unmet needs of people living with diabetes	90	4.37(4.0)	0.60	✓				
Identify and address barriers to engagement	95	4.45(4.5)	0.55	✓				
Promote culture and diversity in health planning	97	4.63(5.0)	0.48	✓				
**PRACTICE LEVEL 4 – PROFICIENT**								
Recognise when there are difficulties engaging with an individual	97	4.71(5.0)	0.43	✓				
**PRACTICE LEVEL 5A – ADVANCED**								
Apply advanced strategies to support engagement	97	4.53(5.0)	0.55	✓				
Reflect on and address barriers to engagement	97	4.63(5.0)	0.48	✓				
Responds professionally to the persons' expression of negative emotions	97	4.74(5.0)	0.40	✓				
Demonstrate self-awareness and caring for oneself	97	4.68(5.0)	0.45	✓				
**PRACTICE LEVEL 5B – ADVANCED**								
Encourage more adaptive behaviours or emotional responses from a person living with diabetes to a given trigger	95	4.55(5.0)	0.54	✓				
Integrate and mentor in principles of cultural competence and diversity in the organisation	97	4.55(5.0)	0.54	✓				
Incorporate self-awareness as a strategic approach to managing oneself and others	97	4.66(5.0)	0.47	✓				
**PRACTICE LEVEL 6 – EXPERT**								
Mentor other health professionals in person-centred care	95	4.68(5.0)	0.47	✓				
Employ self-awareness for effective leadership	97	4.76(5.0)	0.37	✓				
**PRACTICE LEVEL 7 - MASTER**								
Promote guidelines and policy to support therapeutic relationships	95	4.79(5.0)	0.35	✓				

The health professional or health assistant will:

Communicate with influence	Round 1				Round 2			
Capability components	Rank %	Mean (Med)	MAD	Out come	Rank %	Mean (Med)	MAD	Out come
**PRACTICE LEVEL 1 - FOUNDATIONAL**								
Support the person living with diabetes to identify solutions to improve health	50	3.43(3.5)	0.78	R2	50	3.42(3.5)	0.75	**FG-✓**
Value and respect diversity	98	4.60(5.0)	0.50	✓				
Be culturally sensitive	98	4.68(5.0)	0.47	✓				
**PRACTICE LEVEL 2 - PRACTISED**								
Communicate clearly and promote the Diabetes Australia language statement in conversations	84	4.21(4.0)	0.62	✓				

Demonstrate effective communication skills, enabling the health professional to listen, understand and adapt to the audience	97	4.63(5.0)	0.48	✓				
Document precise records to support diabetes care	97	4.68(5.0)	0.45	✓				
Recognise signs of low health literacy and advocate for the persons' healthcare	97	4.58(5.0)	0.51	✓				
**PRACTICE LEVEL 3 – EXPERIENCED**								
Promote healthy conversations with and for people living with diabetes	92	4.45(5.0)	0.58	✓				
Be aware of one's own thoughts, emotions, communication style an boundaries	100	4.68(5.0)	0.43	✓				
Identify and draw attention to important diabetes information to support diabetes care	92	4.34(4.0)	0.55	✓				
Critically appraise health information sources and discuss with the person living with diabetes	87	4.16(4.0)	0.49	✓				

Communicate with influence and leadership	Round 1				Round 2			
Capability components	Rank %	Mean (Med)	MAD	Out come	Rank %	Mean (Med)	MAD	Out come
**PRACTICE LEVEL 4 – PROFICIENT**								
Engage and commit to healthy conversations with and for people living with diabetes	95	4.71(5.0)	0.44	✓				
Demonstrates awareness of one's interaction style & its impact	97	4.74(5.0)	0.40	✓				
**PRACTICE LEVEL 5A – ADVANCED**								
Promote a supportive, open and empathic conversation style	97	4.71(5.0)	0.43	✓				
Document precise, succinct and contextual records to support diabetes care	97	4.79(5.0)	0.34	✓				
Understand and apply learning principles according to the target audience	97	4.71(5.0)	0.43	✓				
**PRACTICE LEVEL 5B – ADVANCED**								
Mentor other health professionals in healthy conversations with and for people living with diabetes	97	4.66(5.0)	0.47	✓				
Advocates for effective communication skills in diabetes care	97	4.71(5.0)	0.43	✓				
Mentor others in record-keeping to improve support for diabetes care	92	4.58(5.0)	0.55	✓				
**PRACTICE LEVEL 6 – EXPERT**								
Integrate evidenced-based knowledge on strategies to support healthy conversations with and for people living with diabetes	97	4.87(5.0)	0.24	✓				
Prepares fact sheets and supportive material in specialty field for diabetes care	76	4.24(4.5)	0.76	✓				
Lead change and endeavours to improve record-keeping and communications to support diabetes care	95	4.63(5.0)	0.50	✓				
**PRACTICE LEVEL 7 - MASTER**								
Demonstrate excellent communication skills when working with people living with diabetes	97	4.89(5.0)	0.19	✓				
Influence and negotiates persuasively	97	4.79(5.0)	0.34	✓				
Inspire and lead with purpose	97	4.76(5.0)	0.37	✓				

The health professional or health assistant will:

Support counselling to achieve agreed outcomes	Round 1				Round 2			
Capability components	Rank %	Mean (Med)	MAD	Out come	Rank %	Mean (Med)	MAD	Out come
**PRACTICE LEVEL 1 - FOUNDATIONAL**								
Support implementing a self-management plan	68	3.70(4.0)	0.81	R2	64	3.72(4.0)	0.74	FG-✓
Identify psychological distress	83	3.95(4.0)	0.53	✓				
**PRACTICE LEVEL 2 - PRACTISED**								
Support the person make changes to fit diabetes into their life	76	4.03(4.0)	0.61	✓				
Identify emotional health	92	4.32(4.0)	0.54	✓				
Demonstrate an understanding of psychosocial impact of living with diabetes	76	4.11(4.0)	0.61	✓				

PRACTICE LEVEL 3 – EXPERIENCED								
Promote implementation and monitoring of personalised self-management plans	82	4.32(4.5)	0.68	✓				
Incorporate counselling techniques and shared decision-making strategies to achieve outcomes	82	4.21(4.0)	0.62	✓				
Monitor for emotional health	90	4.34(4.0)	0.59	✓				
Coordinate care for people with psychosocial impact of living with diabetes	82	4.24(4.0)	0.64	✓				
Advocate for peer support engagement	79	4.05(4.0)	0.50	✓				

Exemplify counselling to achieve agreed outcomes	Round 1				Round 2			
Capability components	Rank %	Mean (Med)	MAD	Out come	Rank %	Mean (Med)	MAD	Out come
**PRACTICE LEVEL 4 – PROFICIENT**								
Tailor personalised diabetes self-management plans	97	4.71(5.0)	0.43	✓				
Counsel with meaning and shared decision-making	95	4.71(5.0)	0.44	✓				
Support connection with peer support groups and services for referral	97	4.66(5.0)	0.47	✓				
**PRACTICE LEVEL 5A – ADVANCED**								
Consult in, develop and monitor personalised diabetes self-management plans in partnership with consumer	97	4.66(5.0)	0.47	✓				
Determine counselling techniques and shared decision-making to achieve outcomes	95	4.47(5.0)	0.55	✓				
Monitors for eating disorders and disordered eating and refers	87	4.26(4.0)	0.58	✓				
Coordinate care for people with evidence of psychosocial impact of living with diabetes	92	4.50(5.0)	0.58	✓				
Enable problem-solving by supporting the individual to identify attitudinal and affective factors which impact self-management behaviours	97	4.55(5.0)	0.52	✓				
**PRACTICE LEVEL 5B – ADVANCED**								
Mentor and consult health professionals to support implementing and monitoring personalised self-management plans	95	4.53(5.0)	0.55	✓				
Mentor other health professionals in counselling techniques	87	4.32(4.5)	0.68	✓				
Lead care coordination for people with symptoms of psychosocial impact of living with diabetes	92	4.32(4.0)	0.61	✓				
**PRACTICE LEVEL 6 – EXPERT**								
Integrate guidelines to support the development and quality monitoring of diabetes self-management plans into the organisation	97	4.68(5.0)	0.45	✓				
Incorporate nuances of counselling techniques related to specialty diabetes	95	4.53(5.0)	0.55	✓				
Incorporate measures and pathways for early screening, detection and care of the psychosocial impact of living with diabetes	97	4.71(5.0)	0.43	✓				
**PRACTICE LEVEL 7 - MASTER**								
Seamlessly apply shared decision-making approach to management choice	97	4.76(5.0)	0.37	✓				
Identifies and diagnoses depression and diabetes-related distress in people living with diabetes	95	4.63(5.0)	0.50	✓				
Leads endeavours to service delivery to reduce the psychosocial impact of diabetes in people living with diabetes	97	4.68(5.0)	0.45	✓				

The health professional or health assistant will:

Support quality use of medicines	Round 1				Round 2			
Capability components	Rank %	Mean (Med)	MAD	Out come	Rank %	Mean (Med)	MAD	Out come
**PRACTICE LEVEL 1 - FOUNDATIONAL**								
Identify *and report* basic issues that can impact on the use of medicines	63	3.63(4.0)	0.87	R2	61	3.64(4.0)	0.94	FG-✓*

**PRACTICE LEVEL 2 – PRACTISED**								
Those intending to be involved in the medicine capability should consider their professional scope of practice, the existence of a regulatory framework for escalation of adverse effects and insurance coverage:								
~~Provide a description of~~ *Describes* basic classes of glucose-lowering medicines available to manage type 2 diabetes	61	3.66(4.0)	0.84	R2	53	3.64(4.0)	0.77	FG-✓*
Identify insulins types and mode of action	68	3.92(4.0)	0.74	R2	70	3.89(4.0)	0.71	FG-✓
Identify and manage issues that can impact on quality use of medicines	74	4.08(4.0)	0.78	✓				
Also, when providing care in partnership with people living with diabetes, authorised registered or medication endorsed nurses or pharmacists will:								
Reinforce education for safe administration of injectable glucose-lowering medicines	84	4.37(5.0)	0.70	✓				
Consider whether the medicine can be administered safely	92	4.58(5.0)	0.60	✓				
Promote and incorporate quality use of medicines principles	92	4.61(5.0)	0.56	✓				
Also, when providing care in partnership with people living with diabetes authorised registered or medication endorsed nurses will:								
Administer glucose-lowering medicines safely	90	4.45(5.0)	0.70	✓				
**PRACTICE LEVEL 3 – EXPERIENCED**								
~~Explain~~ *Recognise* the action of all glucose-lowering medicines available to manage type 2 diabetes	71	3.87(4.0)	0.66	R2	59	3.75(4.0)	0.66	FG--✓*
Promote therapeutic use of glucose-lowering medicines available to manage type 2 diabetes	74	4.03(4.0)	0.72	✓				
Demonstrate understanding of insulins types and mode of action	74	4.03(4.0)	0.67	✓				
Monitor issues that can impact on use of medicines and evaluates responses	76	4.11(4.0)	0.71	✓				
When delivering care to people living with diabetes, all authorised registered or medication endorsed nurses, pharmacists and Diabete Aboriginal and Torres Strait Islander Health Practitioner or Worker will:								
• Educate the person living with diabetes or relevant others in injection technique	79	4.26(4.0)	0.70	✓				
In addition, when providing care in partnership with people living with diabetes, all registered or medication endorsed nurses or pharmacists, endorsed by their organisation will:								
• Reduce medicines risk in people living with diabetes	92	4.34(4.0)	0.55	✓				

**Exemplify quality use of medicines**	**Round 1**				**Round 2**			
**Capability components**	**Rank %**	**Mean (Med)**	**MAD**	**Out come**	**Rank %**	**Mean (Med)**	**MAD**	**Out come**
**PRACTICE LEVEL 4 – PROFICIENT**								
Those intending to be involved in the medicine capability should consider their professional scope of practice, the existence of a regulatory framework for escalation of adverse effects and insurance coverage:								
Understand combinations of glucose-lowering medicines available to manage type 2 diabetes and reduce the risk of adverse effects	97	4.79(5.0)	0.34	✓				
Demonstrate an understanding of insulins regimens to achieve individualised goals	97	4.76(5.0)	0.37	✓				
Promote safe and effective handling and administration of diabetes medicines	97	4.82(5.0)	0.31	✓				
**PRACTICE LEVEL 5A – ADVANCED**								
Support education in and appropriate choice of glucose-lowering medicine for pharmacotherapeutics management of type 2 diabetes	97	4.68(5.0)	0.45	✓				
Support quality use of insulin to manage diabetes	97	4.71(5.0)	0.43	✓				
Understand the place for complementary and alternative medicines in diabetes	79	4.18(4.0)	0.69	✓				

**PRACTICE LEVEL 5B – ADVANCED**								
Incorporate quality use of medicines principles into decision-making	95	4.82(5.0)	0.32	✓				
Promote therapeutic use and monitor the impact of glucose-lowering medicines available to manage type 2 diabetes to ensure safe and effective use	97	4.87(5.0)	0.24	✓				
Optimise insulin therapy in people living with diabetes	92	4.74(5.0)	0.43	✓				
**PRACTICE LEVEL 6 – EXPERT**								
Monitor for the therapeutic use of glucose-lowering medicines available to manage type 2 diabetes	95	4.84(5.0)	0.28	✓				
Monitor for and promote the therapeutic use of insulin to manage type 1 diabetes	95	4.87(5.0)	0.24	✓				
Promote optimisation of diabetes to target through development of novel tools or aides to support management and care	95	4.68(5.0)	0.47	✓				
**PRACTICE LEVEL 7 - MASTER**								
When involved in medicine management while caring for people living with diabetes, the advanced practice CDE who is a 'non-medical prescriber' considering their scope of practice will: Demonstrate proficiency in quality use of medicines	97	4.84(5.0)	0.27	✓				
Meet the National Prescribing Service competencies required to prescribe medicines	97	4.84(5.0)	0.27	✓				
Adeptly choose and prescribe suitable medicines within a defined scope of practice for diabetes care	97	4.84(5.0)	0.27	✓				
Monitor prescribed medicines to optimise diabetes care	97	4.84(5.0)	0.27	✓				
When involved in medicine management while caring for people wit diabetes, the diabetes nurse practitioner will:								
Demonstrate an adept understanding of the quality use of medicines principles	98	4.87(5.0)	0.24	✓				
Proficiently use diabetes and metabolic medicines safely and effectively	95	4.82(5.0)	0.32	✓				
Optimise medicine therapy and minimise drug-related problems	97	4.82(5.0)	0.31	✓				
Monitor the impact of polypharmacy	97	4.76(5.0)	0.37	✓				

The health professional or health assistant will:

Display awareness of diabetes technology	Round 1				Round 2			
**Capability components**	Rank %	Mean (Med)	MAD	Out come	Rank %	Mean (Med)	MAD	Out come
**PRACTICE LEVEL 1 - FOUNDATIONAL**								
Display basic blood glucose and ketone monitoring skills	70	3.98(4.0)	0.84	R2	78	3.94(4.0)	0.70	✓
Recognise technologies used to manage diabetes	40	3.25(3.0)	0.90	R2	39	3.19(3.0)	0.96	x
Be aware of and report basic issues that could impact insulin pump, CGM and Flash Glucose Monitoring technology	38	3.20(3.0)	0.90	x				
**PRACTICE LEVEL 2 - PRACTISED**								
Demonstrate blood glucose and ketone monitoring skills	90	4.53(5.0)	0.62	✓				
~~Show understanding~~ *Recognise* technologies used to manage type 1 diabetes	42	3.37(3.0)	0.74	R2	47	3.42(3.0)	0.69	FG*-✓*
Recognise potential ~~basic~~ *fundamental* problems with insulin pump, or Continuous Glucose or Flash Glucose Monitoring sensor use	42	3.26(3.0)	0.78	R2	45	3.31(3.0)	0.84	FG*-✓*
**PRACTICE LEVEL 3 – EXPERIENCED**								
Demonstrate proficiency at blood glucose and ketone monitoring	90	4.45(5.0)	0.61	✓				
Display awareness of technologies used to manage diabetes	68	3.89(4.0)	0.62	R2	67	3.80(4.0)	0.61	FG -✓
Encourage the consumer to apply problem-solving skills for insulin pump, Continuous Glucose or Flash Glucose Monitoring sensor use	45	3.45(3.0)	0.86	R2<br>EAG Ad	50	3.43(4.0)	0.86	FG -✓*

Advocate for the use of diabetes technology in ~~Aboriginal and Torres Strait Islander people~~ *individuals* with diabetes	55	3.63(4.0)	0.83	R2	61	3.72(4.0)	0.74	**FG -✓***
**PRACTICE LEVEL 4 – PROFICIENT**								
Demonstrate adept skills in blood glucose and ketone monitoring	97	4.79(5.0)	0.34	✓				
Support the use of diabetes technologies to manage diabetes	92	4.53(5.0)	0.60	✓				
Promote problem-solving skills for insulin pump, Continuous or Flash Glucose Monitoring sensor use	84	4.26(4.0)	0.70	✓				

Exemplify quality use of diabetes technology	Round 1				Round 2			
Capability components	Rank %	Mean (Med)	MAD	Out come	Rank %	Mean (Med)	MAD	Out come
**PRACTICE LEVEL 5A – ADVANCED**								
Adeptly promote blood glucose and ketone monitoring and assessing blood glucose variability	97	4.79(5.0)	0.34	✓				
Capable of accessing and reviewing ambulatory glucose profiles (AGP)	95	4.47(5.0)	0.55	✓				
Aid with diabetes technologies to manage diabetes	97	4.45(4.0)	0.52	✓				
Apply problem-solving skills for Continuous and Flash Glucose Monitoring sensor, and insulin pump use	90	4.29(4.0)	0.56	✓				
**PRACTICE LEVEL 5B – ADVANCED**								
Proficiently educate in and commence Flash and Continuous Glucose Monitoring systems	95	4.66(5.0)	0.49	✓				
Comprehend ambulatory glucose profiles and, reviews and analyses Continuous or Flash Glucose Monitoring reports	95	4.63(5.0)	0.50	✓				
Competently educate, commence and monitor Insulin Pump Therapy	79	4.11(4.0)	0.71	✓				
**PRACTICE LEVEL 6 – EXPERT**								
Demonstrate knowledge of contemporary diabetes technological advances	97	4.71(5.0)	0.43	✓				
Comprehensively explain the processes & logistics required to commence a person living with diabetes on an insulin pump	92	4.61(5.0)	0.54	✓				
Adeptly train a person living with type 1 diabetes regarding insulin pump therapy	90	4.50(5.0)	0.61	✓				
Adeptly monitor insulin pump therapy, Continuous and Flash Glucose Monitoring, and interpret ambulatory glucose profiles and reports	97	4.68(5.0)	0.45	✓				
Proficiently provide advice to people using insulin pump therapy to optimise care	97	4.66(5.0)	0.47	✓				
**PRACTICE LEVEL 7 - MASTER**								
Be adept at interpreting ambulatory glucose profiles, and differing Continuous or Flash Glucose Monitoring and pump reports to optimise diabetes care	97	4.79(5.0)	0.34	✓				
Apply comprehensive advanced skills to manage a person living with diabetes using insulin pump therapy	92	4.74(5.0)	0.43	✓				

The health professional or health assistant will:

Support care coordination	Round 1				Round 2			
Capability components	Rank %	Mean (Med)	MAD	Out come	Rank %	Mean (Med)	MAD	Out come
**PRACTICE LEVEL 1 - FOUNDATIONAL**								
Identify internal and external relationships	65	3.73(4.0)	0.61	R2	64	3.83(4.0)	0.88	**FG-✓**
Listen to and understand the needs of others	90	4.35(4.0)	0.59	✓				
Share learnings and support others	83	4.13(4.0)	0.53	✓				
**PRACTICE LEVEL 2 - PRACTISED**								
Maintain accurate and confidential records of clinical care	100	4.89(5.0)	0.19	✓				
Engage in care coordination	97	4.68(5.0)	0.45	✓				

Listen to, understand and recognise the needs of others	100	4.82(5.0)	0.30	✓				
Share learning and supports others	100	4.71(5.0)	0.41	✓				
**PRACTICE LEVEL 3 – EXPERIENCED**								
Support seamless transition through quality record keeping	92	4.63(5.0)	0.62	✓				
Develop care plans and manage care coordination	87	4.34(4.0)	0.62	✓				
Nurture relevant external relationships conducive to care of chronic conditions	90	4.61(5.0)	0.56	✓				
Share learning and support others	92	4.45(5.0)	0.58	✓				

**Lead care coordination**	Round 1				Round 2			
**Capability components**	Rank %	Mean (Med)	MAD	Out come	Rank %	Mean (Med)	MAD	Out come
**PRACTICE LEVEL 4 – PROFICIENT**								
Engage in care coordination	95	4.55(5.0)	0.54	✓				
Participate as an active member of the interdisciplinary team	97	4.79(5.0)	0.34	✓				
**PRACTICE LEVEL 5A – ADVANCED**								
Implement activities and processes that facilitate care coordination	97	4.68(5.0)	0.45	✓				
Promote and engage in interdisciplinary collaborative practice	97	4.74(5.0)	0.40	✓				
**PRACTICE LEVEL 5B – ADVANCED**								
Apply broad clinical and systems approach to improve care coordination	92	4.58(5.0)	0.55	✓				
Cultivate an environment of collaborative practice and acts as a role model	95	4.71(5.0)	0.44	✓				
**PRACTICE LEVEL 6 – EXPERT**								
Expertly tailors care plans and care coordination	97	4.71(5.0)	0.43	✓				
Establish a culture of collaboratively interdisciplinary practice	97	4.84(5.0)	0.27	✓				
Identify barriers and solutions to care coordination in the specialty area	97	4.79(5.0)	0.34	✓				
**PRACTICE LEVEL 7 - MASTER**								
Mentor in care coordination, contribute to planning and policy	94	4.66(5.0)	0.49	✓				
Exemplify care coordination and interdisciplinary collaboration	97	4.82(5.0)	0.31	✓				

The health professional or health assistant will:

**Achieve quality**	Round 1				Round 2			
**Capability components**	Rank %	Mean (Med)	MAD	Out come	Rank %	Mean (Med)	MAD	Out come
**PRACTICE LEVEL 1 - FOUNDATIONAL**								
Support quality improvement	78	3.95(4.0)	0.68	✓				
**PRACTICE LEVEL 2 - PRACTISED**								
Engage in quality improvement	92	4.53(5.0)	0.57	✓				
Responsive to change	97	4.58(5.0)	0.51	✓				
**PRACTICE LEVEL 3 – EXPERIENCED**								
Seek and critically review publications related to diabetes education and care *to inform care*	63	3.89(4.0)	0.71	R2	44	3.35(3.0)	0.94	**FG*-✓***
Engage in quality improvement	90	4.42(5.0)	0.61	✓				
Responsive to change	90	4.47(5.0)	0.61	✓				

**Cultivate quality through leadership and research**	Round 1				Round 2			
**Capability components**	Rank %	Mean (Med)	MAD	Out come	Rank %	Mean (Med)	MAD	Out come
**PRACTICE LEVEL 4 – PROFICIENT**								
Identify relevant evidence-base to guide practice	97	4.74(5.0)	0.40	✓				
Engage in quality improvement	97	4.71(5.0)	0.43	✓				
Responsive to change	97	4.79(5.0)	0.34	✓				

**PRACTICE LEVEL 5A – ADVANCED**					
Support research and quality improvement to build the evidence to promote improvements in self-management of prediabetes and diabetes education and care	95	4.45(4.5)	0.55	✓	
**PRACTICE LEVEL 5B – ADVANCED**					
Develop a culture of quality improvement	97	4.74(5.0)	0.40	✓	
Participate in research and quality improvement to build the evidence to promote improvements in self-management of prediabetes and diabetes education and care	95	4.58(5.0)	0.53	✓	
Embrace change towards improvements in diabetes care	97	4.82(5.0)	0.31	✓	
**PRACTICE LEVEL 6 – EXPERT**					
Cultivate a culture of continuous improvement	97	4.87(5.0)	0.24	✓	
Influence research and quality improvement to build the evidence base to improve lives of people living with diabetes and prediabetes	95	4.71(5.0)	0.44	✓	
**PRACTICE LEVEL 7 - MASTER**					
Apply research proficiently into advanced practice	95	4.71(5.0)	0.44	✓	
Demonstrate a commitment to lifelong learning	97	4.79(5.0)	0.34	✓	
Lead research and drive innovation	94	4.55(5.0)	0.59	✓	

Consensus Outcome Scale:
✓ = accepted
R2 = carried over to Round 2 for ranking
X = rejected
FG = sent to focus group
FG * = sent to focus group on advice of the Expert Advisory Group
FG-X = rejected by focus group
✓ * = accepted by focus group with amendment as marked in italics/strikethrough
FG-Add = additional capability component added by focus group

Level of Agreement:
High =
Moderate =
Low =

Table S7: Minimum level of clinical competence in diabetes care required by roles

Practice Level 1	Practice Level 2	Practice Level 3	Practice Level 4	Practice Level 5	Practice Level 6	Practice Level 7
**Foundational**<br>Certificate II or III	**Practised**<br>Undergraduate degree<br>Advanced Diploma<br>Certificate IV	**Experienced**<br>Bachelor degree	**Proficient**<br>Bachelor degree ± PGC<br>Certificate IV + PGC	**Advanced**<br>Bachelor degree + PGC	**Expert**<br>Bachelor degree + PGD	**Master**<br>Bachelor degree + PGD ± Masters<br>Masters
• Assistant in Nursing<br>• Personal Care Assistant<br>• Allied Health Assistant<br>• Personal Carer<br>• Health Care Assistant<br>• Pharmacy Assistant<br>• Aboriginal and Torres Strait Islander Health Practitioner [PT]	• Generalist nurse<br>• Generalist AHP<br>• Midwife [PT]<br>• Enrolled Nurse<br>• Aboriginal and Torres Strait Islander Health Practitioner [PT] (AHPRA registered)	• Primary healthcare nurse<br>• Practice Nurse<br>• Primary healthcare AHP<br>• Community Nurse	• Diabetes Educator (Nurse or AHP)<br>• Aboriginal and Torres Strait Islander ***Diabetes*** Health Practitioner	• Credentialled Diabetes Educator [TM] [*Novice (5A)-*Experienced (5B)] – Nurse or AHP	• Specialist Credentialled Diabetes Educator [*Expert] – Nurse or AHP	• Nurse Practitioner [PT]

*Aligned with ADEA 3 levels of competence for a CDE
Does not constitute a title but is a descriptor

PGD=Postgraduate diploma
PGC=Postgraduate certificate

NB: Advanced Management Credentialled Diabetes Educator with non-medical prescriber endorsement recommended and thought to sit in practice level 6 or 7.
